# Individual- and Group-Level Disparities Between Racial and Ethnic Groups in Lung Cancer Screening Eligibility Criteria

**DOI:** 10.1001/jamanetworkopen.2025.2172

**Published:** 2025-03-27

**Authors:** Corey D. Young, Hormuzd A. Katki, Li C. Cheung, M. Patricia Rivera, Hilary A. Robbins, Melinda C. Aldrich, Jeffrey D. Blume, Anil K. Chaturvedi, Rebecca Landy

**Affiliations:** 1 Division of Cancer Epidemiology and Genetics, National Cancer Institute, National Institutes of Health, Department of Health and Human Services, Bethesda, Maryland; 2 Division of Pulmonary and Critical Care Medicine, University of Rochester Medical Center, Rochester, New York; 3 Genomic Epidemiology Branch, International Agency for Research on Cancer, Lyon, France; 4 Department of Medicine, Division of Genetic Medicine, Vanderbilt University Medical Center, Tennessee; 5 School of Data Science, University of Virginia, Charlottesville

## Abstract

**Importance:**

Lung cancer screening guidelines result in differential screening eligibility among individuals who might benefit equally from screening and in population-level differences in screening eligibility and benefit across races and ethnicities.

**Objective:**

To inform lung cancer screening policy development by evaluating how enforcing (1) equal lung cancer screening eligibility for all individuals with equal benefit and (2) equal program sensitivity across racial and ethnic subgroups are associated with screening eligibility and benefit.

**Design, Setting, and Participants:**

This cross-sectional comparative effectiveness study included 6915 members of the US noninstitutionalized population aged 50 to 80 years who ever smoked and who participated in the 2015 National Health Interview Survey. Statistical analysis was performed from May 2022 to April 2024.

**Exposure:**

Lung cancer screening eligibility is based on the LYFS-CT (life-years gained from screening–computed tomography) prediction model, which predicts gain in life expectancy from screening, where individuals are eligible if their predicted benefit exceeds a threshold across all possible thresholds.

**Main Outcomes and Measures:**

The proportion of individuals aged 50 to 80 years who ever smoked who are eligible for screening, the percentage of predicted gainable life gained from screening (program sensitivity), and the number needed to screen to gain 10 years of life (screening efficiency), by race and ethnicity.

**Results:**

The 6915 participants aged 50 to 80 years who ever smoked represented 44 million individuals (mean age, 63 years [IQR, 56-69 years]; 53% male; 68% formerly smoked; 10% African American individuals, 3% Asian American individuals, 8% Hispanic American individuals, and 79% non-Hispanic White individuals). To ensure equal screening eligibility for each race and ethnicity required race- and ethnicity-specific eligibility thresholds. To achieve 36% eligibility for each race and ethnicity, the required days of life gained (under the LYFS-CT model) screening eligibility thresholds would be 5.2 for Hispanic American individuals, 5.6 for Asian American individuals, 9.5 for non-Hispanic White indivduals, and 12.4 for African American individuals, so individuals of different races and ethnicities with the same benefit would have different eligibility. With a fixed eligibility threshold of 16.2 days, screening eligibility would differ across races and ethnicities; 7% of Hispanic American individuals, 9% of Asian American individuals, 20% of non-Hispanic White individuals, and 27% of African American individuals aged 50 to 80 years who ever smoked would be eligible for screening. Similar differences existed for the program sensitivity of screening benefit. African American individuals consistently maintained the most efficient number needed to screen across all thresholds; Hispanic American individuals had the least efficient number needed to screen and thus may experience the worst benefit-harm balance when equalizing program sensitivity between races and ethnicities.

**Conclusions and Relevance:**

This comparative effectiveness study of lung cancer screening eligibility suggests that screening eligibility criteria cannot result in both equal eligibility for all individuals with the same benefit and equal program sensitivity for each race and ethnicity. In general, race- and ethnicity-specific thresholds that result in equal group-level sensitivity on 1 metric cannot result in equal sensitivities on other metrics. Thus, only 1 metric can be equalized, requiring a value judgment on which to prioritize.

## Key Points

**Question:**

How do lung cancer screening eligibility and associated benefits differ when policymakers prioritize (1) equal percentage eligibility or benefit across self-reported racial and ethnic subgroups and (2) equal individual-level eligibility criteria based on estimated screening benefit?

**Findings:**

This comparative effectiveness study found that achieving equal eligibility across racial and ethnic groups required race-specific screening thresholds, leading to differential eligibility for individuals with the same predicted benefit. With a fixed eligibility threshold, screening eligibility and benefit varied across racial and ethnic groups.

**Meaning:**

This study suggests that the target metric of a screening intervention should be explicitly identified for equity considerations, because there is often a trade-off between equalizing eligibility or benefit across groups and equalizing the opportunity to be screened for all individuals with the same estimated benefit (using the chosen benefit metric).

## Teaser

This comparative effectiveness study informs lung cancer screening policy development by evaluating how enforcing (1) equal lung cancer screening eligibility for all individuals with equal benefit and (2) equal program sensitivity across racial and ethnic subgroups are associated with screening eligibility and benefit.

## Introduction

Lung cancer screening has been recommended in the US since 2013, based on results from the National Lung Screening Trial, which showed a 20% reduction in 5-year lung cancer mortality among individuals invited for 3 annual low-dose computed tomography (CT) screenings compared with chest radiographs.^1^ The United States Preventive Services Task Force (USPSTF) 2013 guidelines recommended screening individuals aged 55 to 80 years with 30 or more pack-years who either currently smoked or had quit within the past 15 years.^2^ However, these guidelines resulted in racial and ethnic inequities, with differences in the proportion of individuals with lung cancer eligible for screening (program sensitivity), as well as the proportion of individuals aged 55 to 80 years who ever smoked who would be eligible for screening.^3,4,5,6^ Aldrich et al^4^ showed that a race- and ethnicity-specific criterion of 20 or more pack-years for African American individuals and 30 or more pack-years for White individuals reduced the difference in sensitivity between African American and White individuals. Lowering the age criteria to 50 years for African American individuals would further reduce this difference.^4^

In 2021, the USPSTF updated its recommendations, lowering the pack-year criteria to 20 or more for all races and ethnicities and expanding the age range to 50 to 80 years.^7,8^ The 2021 recommendations state that “Screening for lung cancer in persons at an earlier age and with fewer pack-years of smoking (ie, 20 pack-years) may also help partially ameliorate racial disparities in screening eligibility,”^8^ and the USPSTF recently committed to ensuring its recommendations reduce disparities.^9^ However, a modeling study led by our group suggested that relaxing eligibility criteria for all races and ethnicities is not associated with a reduction in the absolute differences in the proportion of lung cancer deaths prevented or life gained between White individuals and individuals of any racial or ethnic minority group (using the 2021 guidelines compared with 2013 recommendations).^10^ In addition, individuals at equal risk or benefit (estimated using prediction models) have different eligibility under the 2021 USPSTF guidelines.

One way to reduce racial and ethnic disparities in lung cancer screening eligibility is to use prediction models to inform eligibility,^5,10,11,12,13,14,15,16^ and both the National Comprehensive Cancer Network^17^ and the American College of Chest Physicians (ACCP)^18^ recommend the use of prediction models for assessing screening eligibility. The use of race and ethnicity in clinical prediction models (“race-aware models”) is controversial, although it is likely required in models informing lung cancer screening to improve existing disparities.^19^ Assuming that prediction models performed perfectly, and therefore equally well for everyone (which is not the current situation^20,21^), and that health care access and delivery were equal for everyone, using a single threshold to assess eligibility would ensure equal screening eligibility for all individuals with respect to 1 metric. For example, if equal screening eligibility was desired with respect to lung cancer mortality, then using lung cancer mortality risk to assess screening eligibility would ensure that everyone with the same risk would have the same screening eligibility. Individual-level equality is the same concept as “horizontal equity” as used by Sasieni,^22^ with group-level equality equivalent to “vertical equity.”

When considering disparities in screening eligibility, it is crucial to consider the harms in addition to the benefits associated with screening and to take both a population and individual perspective. For example, in a hypothetical scenario, cancer cases might occur almost exclusively among individuals at high risk in group A, while cases are distributed across individuals at lower and medium risk in group B, potentially due to differences in behavioral risk factors or performance of the risk model (ie, underestimating risk among individuals in group B).^23^ Eligibility criteria could be chosen to ensure that the same percentage of lung cancer cases (or individuals) are eligible for screening in every group, achieving equal program sensitivity in that metric across groups at the population level. However, when considering the individuals in each group, many individuals in group B would be eligible for screening, despite their lower risk, and would thus be likely to experience more harms than benefits from screening. Some harms of lung cancer screening are experienced on an individual level, including diagnostic workup after false-positive screening results, as well as psychological harm^24^; other harms can only be quantified on a population level, such as radiation-induced cancers and overdiagnosis and overtreatment. In addition, the 20% reduction in lung cancer mortality from screening is a population-level benefit; each individual either does or does not have their life saved by screening, and it is not possible to know which individuals’ lives have been saved.

Many possible metrics can be used to assess whether screening eligibility criteria result in disparities, such as the proportion of individuals who are eligible for screening or the proportion of individuals with lung cancer who would have been eligible for screening. To assess disparities and create guidelines that eliminate disparities, the most relevant metrics of disparity must be identified because it is often not possible to ensure that all metrics of interest will be equal with a single screening strategy. The 2021 USPSTF guidelines^8^ refer to disparities in the proportion of individuals who ever smoked who were eligible for screening and in the proportion of people who developed lung cancer who were eligible for screening, 2 measures of program sensitivity. However, especially when considering screening an asymptomatic population, we must also appropriately weigh the harms of screening, with, for example, screening efficiency (the number of people who need to be screened to benefit a single person).

To inform organizations that set lung cancer screening policies, we use nationally representative data to consider the impact of assigning screening eligibility based on the concepts of equal outcomes on a population level across racial and ethnic categories and equal eligibility on an individual level of individuals with the same risk or benefit. We consider eligibility metrics, the proportion of gainable life (ie, life gained from screening everyone) that would be gained by screening eligible individuals (program sensitivity), and screening efficiency. Life gained was selected instead of lung cancer incidence or mortality because the main goal of screening is to extend life. The benefit associated with screening individuals at high risk of competing mortality may be reduced if there are greater harms from surgical procedures or an increased risk of death from other causes even when lung cancer death is prevented. However, many of the individuals with the highest lung cancer risk are also the individuals at highest risk of competing mortality.^25^

## Methods

### LYFS-CT Prediction Model

For this study, our screening strategy is based on the LYFS-CT (life-years gained from screening–computed tomography) prediction model.^13^ This model estimates the life gained from National Lung Screening Trial (NLST)–like CT screening (3 annual low-dose CT screenings with 5 years of follow-up) by calculating the reduction in lung cancer death risk resulting from screening, combined with life expectancy.^13^ This approach maximizes the benefits associated with screening while reducing harms and is recommended by the ACCP to inform screening eligibility.^18^ Details, including the variables in the LYFS-CT model, have been published^13^ and are provided in the eMethods in Supplement 1, while the models are accessible via our *lcmodels* R package (R Project for Statistical Computing).^26^ The National Institutes of Health Office of Human Subjects Research deemed this study exempt from institutional review board approval under pre–common rule exemption categories because we did not collect any new data. All National Health Interview Survey (NHIS) participants provided informed consent. This study follows the International Society for Pharmacoeconomics and Outcomes Research (ISPOR) reporting guidelines for comparative effectiveness research.^27^

### Participants

We applied the LYFS-CT model to individuals aged 50 to 80 years in the 2015 NHIS who had ever smoked. The sample is representative of noninstitutionalized persons in the US after application of sampling weights.^28^ Data from 2015 were used because this is the most recent year with complete smoking data (including cigarettes per day) for individuals who formerly smoked. We restricted our analyses to African American, Asian American, Hispanic American, and non-Hispanic White individuals because only 0.5% of individuals aged 50 to 80 years who had ever smoked were from other racial and ethnic groups. Race and ethnicity were self-reported and were collected according to the Office of Management and Budget’s 1997 Revisions to the Standards for the Classification of Federal Data on Race and Ethnicity.

### Statistical Analysis

Statistical analysis was performed from May 2022 to April 2024. For each potential eligibility threshold for the LYFS-CT model, we identified the US population of individuals who would be eligible for screening. We used previously published methods to empirically model the performance of NLST-like screening.^12^ We assumed that the observed reduction in lung cancer mortality risk (20.4% over 5 years) and the increase in lung cancer detection in the NLST (12.4% over 5 years) applied to all eligible individuals, regardless of race and ethnicity.^16^ Multiple imputation was used to account for missing data; see eMethods in Supplement 1 for details.

Descriptive statistics of the population of individuals aged 50 to 80 years who ever smoked were calculated by race and ethnicity. For each race and ethnicity, at each LYFS-CT model benefit threshold, we calculated (1) the percentage of gainable life that would be gained by screening eligible individuals (program sensitivity), (2) the percentage of individuals aged 50 to 80 years who ever smoked who would be eligible for screening (eligibility), (3) the number needed to screen per 10 years of life gained (screening efficiency), and (4) the number needed to screen per 10 years of life gained by race- and ethnicity-specific decile of life gained from screening, among individuals aged 50 to 80 years who ever smoked. The deciles are “relative” (ie, decile 1 contains people who contribute the top 10% of life gained when the distribution of life gained is considered separately for each race and ethnicity). We then considered how imposing equal program sensitivity for each race and ethnicity, first for eligibility, then for life gained, would be associated with the race- and ethnicity-specific eligibility thresholds required to achieve this. Analyses were carried out in R, version 4.2,^29^ using *lcmodels* (R Project for Statistical Computing).^26^

## Results

The 2015 NHIS reports 6915 participants aged 50 to 80 years who ever smoked, representing 44.0 million people. The mean age was 63 years (IQR, 56-69 years); 53% were male, and 47% were female; 68% formerly smoked; and 10% were African American, 3% were Asian American, 8% were Hispanic American, and 79% were non-Hispanic White) (Table). The sex distribution of individuals aged 50 to 80 years who ever smoked differed by race and ethnicity, with 23% of Asian American individuals aged 50 to 80 years who ever smoked being female vs 48% for African American individuals and non-Hispanic White individuals. African American individuals had the highest proportion of current smoking (42% vs 29%-32% for the other racial and ethnic groups). Among both those currently smoking and those who formerly smoked, non-Hispanic White individuals had the lowest proportion of individuals who had smoked less than 20 pack-years (currently smoking: 34% for non-Hispanic White individuals vs 70% for African American individuals; formerly smoked: 59% for non-Hispanic White individuals vs 74% for Hispanic American individuals) and the highest proportion who had smoked 50 or more pack-years.

**Table.** Key Characteristics of Individuals Aged 50 to 80 Years Who Ever Smoked in the 2015 National Health Interview Survey

Characteristic	No. (%)^a^				
	African American (n = 4 333 903)	Asian American (n = 1 266 935)	Hispanic American (n = 3 569 321)	Non-Hispanic White (n = 34 868 520)	Total (N = 44 038 679)
Sex					
Female	2 083 909 (48)	292 576 (23)	1 352 899 (38)	16 825 476 (48)	20 554 860 (47)
Male	2 249 994 (52)	974 360 (77)	2 216 422 (62)	18 043 044 (52)	23 483 820 (53)
Age, mean (IQR), y	62 (55-67)	61 (54-68)	61 (54-67)	63 (56-69)	63 (56-69)
Age, y					
50-54	923 166 (21)	336 948 (27)	908 385 (25)	6 853 167 (20)	9 021 667 (20)
55-59	947 207 (22)	275 759 (22)	809 166 (23)	7 143 614 (20)	9 175 746 (21)
60-64	968 945 (22)	195 636 (15)	631 597 (18)	6 344 152 (18)	8 140 330 (18)
65-69	680 925 (16)	189 968 (15)	573 324 (16)	6 253 658 (18)	7 697 875 (17)
70-74	465 795 (11)	192 381 (15)	340 071 (10)	4 710 511 (14)	5 708 758 (13)
75-80	347 865 (8)	76 242 (6)	306 779 (9)	3 563 418 (10)	4 294 304 (10)
Smoking status					
Currently smoking	1 839 425 (42)	369 682 (29)	1 149 043 (32)	10 768 669 (31)	14 126 819 (32)
Formerly smoked	2 494 478 (58)	897 254 (71)	2 420 278 (68)	24 099 851 (69)	29 911 861 (68)
Quit years (% of former smokers)					
<1	112 722 (5)	21 490 (2)	65 937 (3)	742 651 (3)	942 800 (3)
1-4	318 222 (13)	127 388 (14)	173 113 (7)	2 172 354 (9)	2 791 077 (9)
5-9	201 692 (8)	45 668 (5)	192 606 (8)	2 386 805 (10)	2 826 772 (9)
10-19	526 726 (21)	241 520 (27)	569 764 (24)	4 087 667 (17)	5 425 677 (18)
20-29	697 859 (28)	181 148 (20)	596 989 (25)	5 207 430 (22)	6 683 426 (22)
≥30	637 256 (26)	280 040 (31)	821 869 (34)	9 502 945 (39)	11 242 110 (38)
Current smokers, pack-years (% of current smokers)					
<20	1 110 003 (60)	259 481 (70)	799 515 (70)	3 679 982 (34)	5 848 981 (41)
20-29	346 175 (19)	46 574 (13)	176 793 (15)	2 109 486 (20)	2 679 027 (19)
30-49	256 395 (14)	57 806 (16)	137 210 (12)	3 383 041 (31)	3 834 452 (27)
50-69	89 603 (5)	4097 (1)	21 061 (2)	951 430 (9)	1 066 192 (8)
≥70	37 249 (2)	1723 (0)	14 465 (1)	644 730 (6)	698 167 (5)
Former smokers, pack-years (% of former smokers)					
<20	1 810 667 (73)	658 595 (73)	1 779 272 (74)	14 191 728 (59)	18 440 262 (62)
20-29	254 726 (10)	81 410 (9)	238 736 (10)	3 268 060 (14)	3 842 932 (13)
30-49	269 683 (11)	126 648 (14)	232 058 (10)	4 129 444 (17)	4 757 833 (16)
50-69	100 166 (4)	18 498 (2)	114 977 (5)	1 294 593 (5)	1 528 234 (5)
≥70	59 235 (2)	12 103 (1)	55 236 (2)	1 216 025 (5)	1 342 599 (4)

^a^ Unweighted numbers: African American individuals, 4583; Asian American individuals, 945; Hispanic American individuals, 3157; non-Hispanic White individuals, 25 888.

### 2021 USPSTF Guidelines

Under the 2021 USPSTF guidelines, 14.5 million individuals (33%) were eligible for lung cancer screening. The benefit program sensitivity is not equal across all races and ethnicities because a different proportion of gainable life is gained among each race and ethnicity, ranging from 37% for Hispanic American individuals to 65% for non-Hispanic White individuals (eTable 1 in Supplement 1). Screening efficiency also varied between races and ethnicities (eTable 1 in Supplement 1), with the lowest number needed to screen per 10 years of life gained for African Americans individuals (n = 159), vs 322 for Hispanic American individuals. However, many individuals at high risk and at high benefit are excluded from screening under the 2021 USPSTF guidelines (eResults in Supplement 1).

### Screening Eligibility Based on Individual-Level Benefit

**Figure 1.**
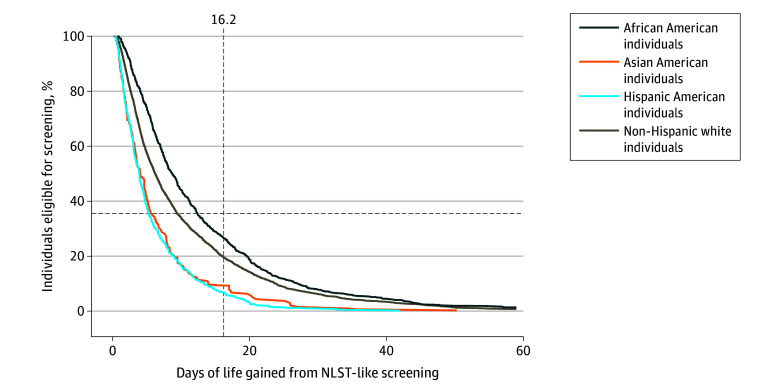
Percentage of Individuals Aged 50 to 80 Years Who Ever Smoked and Who Are Eligible for Screening at Each Screening Eligibility Threshold Defined by Life Years Gained From Screening–Computed Tomography, by Race and Ethnicity The dashed vertical line shows the situation in which everyone with the same estimated benefit associated with screening is eligible regardless of their race and ethnicity; however, this results in different percentages of individuals aged 50 to 80 years who ever smoked being eligible for screening across races and ethnicities, as demonstrated by the dashed vertical line at 16.2 days of life gained: 7% of Hispanic American individuals, 9% of Asian American individuals, 20% of non-Hispanic White individuals, and 27% of African American individuals aged 50 to 80 years who ever smoked would be eligible for screening. Horizontal lines show the situation when the same percentage of individuals aged 50 to 80 years who ever smoked are eligible for screening in all races and ethnicities, although with different thresholds for screening eligibility between races and ethnicities; for example, at 36% eligibility, indicated by the horizontal dashed line, the required days of life gained thresholds would be 5.2 for Hispanic American individuals, 5.6 for Asian American individuals, 9.5 for non-Hispanic White individuals, and 12.4 for African American individuals. NLST indicates National Lung Screening Trial.

**Figure 2.**
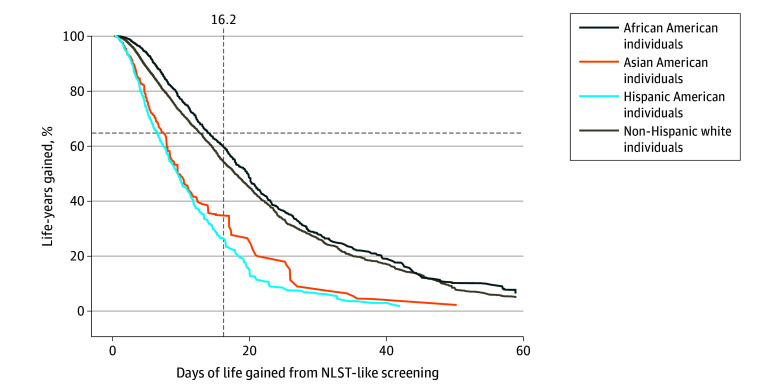
Percentage of Gainable Life That Would Be Gained at Each Screening Eligibility Threshold Defined by Life Years Gained From Screening–Computed Tomography, by Race and Ethnicity The dashed vertical line shows the situation in which everyone with the same benefit associated with screening is eligible regardless of their race and ethnicity; however, this results in different percentages of gainable life being gained among individuals eligible for screening across races and ethnicities, as demonstrated by the dashed vertical line at 16.2 days of life gained: 26% for Hispanic American individuals, 35% for Asian American individuals, 54% for non-Hispanic White individuals, and 60% for African American individuals. Horizontal lines show the situation when the same percentage of gainable life is gained among individuals eligible for screening in all races and ethnicities, although with different thresholds for screening eligibility between races and ethnicities; for example, to gain 65% of gainable life, indicated by the horizontal dashed line, the required days of life gained thresholds would be 6.5 for Hispanic American individuals, 7.3 for Asian American individuals, 12.6 for non-Hispanic White individuals, and 13.8 for African American individuals. NLST indicates National Lung Screening Trial.

We first considered a screening eligibility threshold of 16.2 days of life gained from receiving screening (estimated by the LYFS-CT model), as recommended by the ACCP; in this situation, individuals are screened based only on their estimated benefit. Under this scenario, 7% of Hispanic American individuals, 9% of Asian American individuals, 20% of non-Hispanic White individuals, and 27% of African American individuals aged 50 to 80 years who ever smoked would be eligible for screening (Figure 1; ie, screening eligibility would differ across races and ethnicities). This threshold would also result in considerable group-level differences in the percentage of gainable life that would be gained by screening everyone estimated to gain 16.2 or more days of life (program sensitivity): 26% for Hispanic American individuals, 35% for Asian American individuals, 54% for non-Hispanic White individuals, and 60% for African American individuals (Figure 2).

### Screening Eligibility Based on Group-Level Metrics: Proportion Eligible for Screening

An alternative to managing all individuals the same based on their estimated benefit associated with screening (ie, eligible if their estimated life gained from receiving screening was ≥16.2 days of life, otherwise ineligible) is to make the proportion of individuals aged 50 to 80 years who ever smoked who are eligible for screening the same across each race and ethnicity. To achieve this, each race and ethnicity would need their own benefit threshold to assess eligibility. To achieve 36% eligibility for each race and ethnicity (the proportion of non-Hispanic White individuals eligible under the 2021 USPSTF guidelines), the threshold of required days of life gained would be 5.2 for Hispanic American individuals, 5.6 for Asian American individuals, 9.5 for non-Hispanic White individuals, and 12.4 for African American individuals (Figure 1). Using these thresholds, which lead to the same percentages of each race and ethnicity who are eligible for screening, would yield more comparable program sensitivities in life gained, from 70% for African Americans to 74% for non-Hispanic Whites (Figure 2).

### Eligibility for Screening Based on Group-Level Metrics: Program Sensitivity (Proportion of Gainable Life Gained)

To achieve a program sensitivity of 65% (the program sensitivity among non-Hispanic White individuals under the 2021 USPSTF guidelines), thresholds of 6.5 days would be required for Hispanic American individuals, 7.3 days would be required for Asian American individuals, 12.6 days would be required for non-Hispanic White individuals, and 13.8 days would be required for African American individuals (Figure 2). Thus, defining eligibility based on a group-level metric would result in different screening eligibility for individuals with the same predicted benefit.

**Figure 3.**
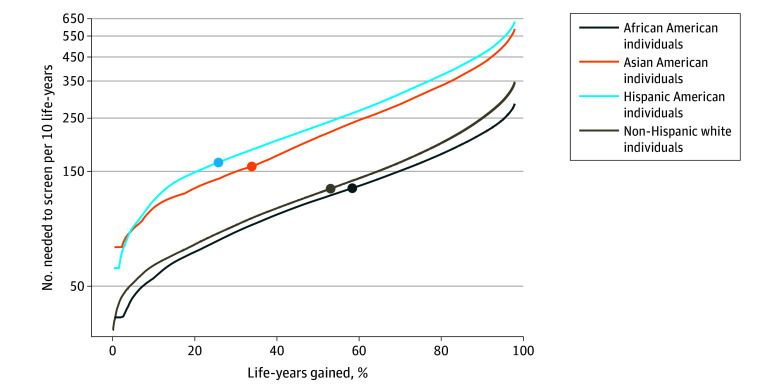
Number Needed to Screen Per 10 Years of Life Gained by Percentage of Gainable Life Gained Among Individuals Aged 50 to 80 Years Who Ever Smoked Circles indicate screening characteristics under the equal scenario using an eligibility threshold of 16.2 days of life gained.

**Figure 4.**
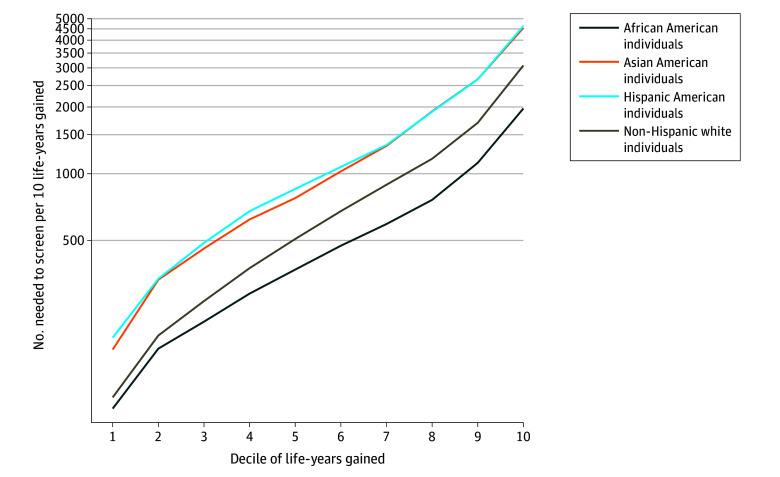
Number Needed to Screen Per 10 Years of Life Gained by Race-Specific Decile of Life Gained From Screening Among Individuals Aged 50 to 80 Years Who Ever Smoked

These differences occur because of the differences in predicted benefit (life gained) distributions between each race and ethnicity (eTable 2 in Supplement 2). This also results in variation in screening efficiency between races and ethnicities (Figure 3). When the percentage of gainable life gained from screening each race and ethnicity is the same, screening is most efficient for African American individuals and is much less efficient for Asian American and Hispanic American individuals. Using the race- and ethnicity-specific thresholds to achieve 65% program sensitivity to life gained, 285.2, 258.9, 148.3, and 138.4 individuals would need to be screened per 10 years of life gained for Hispanic American individuals, Asian American individuals, non-Hispanic White individuals, and African American individuals, respectively (lower numbers correspond to higher efficiency). Alternatively, at a threshold of 16.2 days of life gained from receiving screening, screening efficiency would be 163.6 for Hispanic American individuals, 157.5 for Asian American individuals, 127.3 for non-Hispanic White individuals, and 127.9 for African American individuals. Among each race- and ethnicity-specific decile of benefit, screening was more than twice as efficient for African American individuals compared with Asian American and Hispanic American individuals (Figure 4). As anticipated, screening efficiency decreased in each successive decile as individuals with lower estimated benefit were screened.

## Discussion

The 2021 USPSTF lung cancer screening guidelines result in racial and ethnic disparities in screening eligibility and life gained on both an individual and group level.^14,30,31^ Individuals with the same estimated lung cancer mortality risk and life gained from screening have different screening eligibility. Moreover, the proportion of individuals aged 50 to 80 years who ever smoked and are eligible for screening, as well as the percentage of gainable life achieved among eligible individuals, differ across racial and ethnic groups. However, we have demonstrated that it is not possible both to remove differences in program sensitivity between racial and ethnic groups and to treat all individuals with the same estimated risk or benefit in the same way (ie, either eligible or ineligible for screening). If the same benefit threshold is applied to assess screening eligibility for everyone, differences across racial and ethnic groups remain for both screening eligibility and life gained. Similarly, if the goal is to achieve equal program sensitivities across all racial and ethnic groups on any 1 metric, the threshold to assess eligibility must vary between races and ethnicities, resulting in individuals with the same predicted benefit having different screening eligibility.

Although differences in performance of prediction models between races and ethnicities^20,21^ may play a role, the underlying reason lung cancer screening guidelines cannot achieve equality in both eligibility and benefit associated with screening at the individual and group level is because the underlying predicted benefit distributions differ between each race and ethnicity. Therefore, to identify the same proportion of life gained from screening among each race and ethnicity, a different proportion of individuals aged 50 to 80 years who ever smoked must be eligible, and a different benefit threshold for each race and ethnicity would be required to assess screening eligibility. Assessing eligibility using the LYFS-CT model benefits African American individuals the most because their benefit associated with screening exceeds that of other races and ethnicities. Conversely, fewer Asian American and Hispanic American individuals are estimated to benefit from screening.

The difference in predicted benefit distributions also results in the different number of people of each race and ethnicity who need to be screened to gain 10 years of life from screening, a measure of the harms of screening. Individuals with lower benefit can still experience the harms; we therefore need to consider both the population-level and individual-level perspectives with screening. Although using race- and ethnicity-specific eligibility thresholds can provide equal program sensitivities at a population level, this may increase the harms that individuals with lower benefit would experience. Regardless of whether eligibility thresholds are based on individual-level or group-level metrics, the accuracy of the underlying prediction models is typically not equal for all racial and ethnic groups. This is a requirement for ensuring equality in the desired metric in practice. The 2021 USPSTF guidelines recommended against using prediction models due to insufficient evidence showing this would improve outcomes relative to current guidelines.^8^ However, tools such as Decision Precision+ have been developed to help clinicians implement model-based screening.^32^

The inclusion of race and ethnicity in clinical prediction models is controversial, with many examples of inappropriate use of race and ethnicity in models resulting in reduced access to health care.^33,34,35^ However, studies have also shown that removing race and ethnicity from models results in worse model calibration and less-accurate predictions for individuals from racial and ethnic minority groups.^20,36^ We acknowledge that race and ethnicity are a social construct and that, ideally, race and ethnicity would not need to be included in prediction models if we knew and could include the latent variables that race and ethnicity capture in the model; however, that is not currently possible. Social determinants of health are associated with the risk of lung cancer death and the benefit from screening through their effect on many structural factors (eg, health care access, neighborhood, built environment, education, and economic stability), influencing individuals’ downstream risks.^37^ As defined by the recent Agency for Healthcare Research and Quality Health Equity Methods Project for the USPSTF,^38^ our work relates to “fairness,” the “downstream outcomes and whether algorithmic decisions create discriminatory or unjust impacts in different populations.” Both the USPSTF discussion guide for considering fairness when making recommendations involving clinical prediction models^38^ and its health equity framework^39^ are important resources for guideline committees, as is the 2024 National Academies of Science, Engineering, and Medicine report “Race and Ethnicity in Biomedical Research.”^37^

This issue of removing disparities from cancer screening is not unique to lung cancer screening, nor to race and ethnicity; other examples include gender differences in fecal immunochemical test screening for colorectal cancer and the thresholds triggering a colonoscopy referral.^22^ Our findings emphasize the impossibility of both having the same screening eligibility for all individuals with the same risk or benefit and achieving the same program sensitivity across each racial and ethnic group. Instead, a value judgment must be used when assessing screening eligibility: Is it preferable for all individuals with the same risk or benefit to have the same eligibility, or is it preferable for the group-level sensitivity to be the same for all races and ethnicities? We believe this judgment should be made by screening guideline committees, with the awareness that it is not possible to achieve both. Even if the program sensitivity of screening were the same across races and ethnicities on a population level, this would not necessarily be the case within a US state or within a subgroup, such as rural populations, because predicted benefits are not evenly distributed within members of 1 race or ethnicity across other characteristics.

### Strengths and Limitations

Our study has several strengths, including its use of nationally representative data from the 2015 NHIS and the application of validated prediction models. We considered multiple metrics for evaluating disparities, showing the implications when evaluating eligibility based on individual-level risk or benefit, then based on group-level sensitivity.

Our study also has some limitations. Our findings are based on empirical modeling of short-term outcomes from an NLST-like screening program, not directly observed outcomes. Individuals of different ages or screening histories will receive different numbers of screenings, which may affect their lifetime benefit associated with screening. We assumed that screening prevented 20% of lung cancer deaths that would have occurred in the absence of screening for individuals of all races and ethnicities who ever smoked^16^ and, therefore, that screening compliance mimics that observed in the NLST. This assumption is supported by the lack of statistical heterogeneity in the relative reduction in lung cancer mortality for low-dose CT scan vs chest radiography in the NLST.^16^ Changes in screening since the NLST, such as different nodule management guidelines, may affect the performance of screening.^40,41,42^ In addition, we assumed that the LYFS-CT model predicts the benefit associated with screening accurately for all individuals, although the 2 submodels that the LYFS-CT model comprises both have worse calibration for Asian American individuals compared with non-Hispanic White individuals.^20^ We assume that everyone above the eligibility threshold (regardless of whether the threshold is the same for all races and ethnicities) attends screening. Screening uptake is currently around 20% among eligible individuals.^43^ This will affect equality in outcomes, such as life gained from screening, although equality in screening eligibility could be achieved. Although these limitations may affect the numbers used in our examples, the exact numbers are not key to our central argument.

## Conclusions

In this comparative effectiveness study of lung cancer screening eligibility, we showed that lung cancer screening guidelines cannot both make the eligibility of all individuals with the same estimated risk or benefit associated with screening the same and achieve equal program sensitivity across all races and ethnicities; a policy judgement is required to determine which is preferable. Equal program sensitivity across racial and ethnic groups would require race- and ethnicity-specific thresholds, although this could result in harm at the individual level in lower-benefit racial and ethnic groups. We recommend that organizations proposing screening guidelines explicitly state which metrics of disparities they are prioritizing when setting health policy recommendations.

## Notes

Supplement 1.
eMethods.

eResults.
**eTable 1.** The Percentage of Individuals Aged 50-80 Years Who Ever Smoked Who Would Be Eligible for Screening Under USPSTF-2021 Lung Cancer Screening Eligibility Guidelines, Life-Years Gained Sensitivity for Individuals Eligible Under USPSTF-2021, and the Efficiency of Screening Individuals Eligible Under USPSTF-2021, by Self-Reported Race and Ethnicity **eTable 2.** Mean, 25^th^, 50^th^ and 75^th^ Percentile of Life-Years Gained From Attending Screening, Estimated by the LYFS-CT Model, by Self-Reported Race And Ethnicity **eTable 3.** The LYFS-CT Submodels for Lung Cancer Death and Overall Mortality **eTable 4.** Characteristics of Hypothetical Individuals Who Are High Risk and/or High Benefit but Not Eligible for Lung Cancer Screening Under USPSTF-2021, or Low Risk and/or Low Benefit and Are Eligible for Lung Cancer Screening Under USPSTF-2021
eReferences.

Supplement 2.
Data Sharing Statement
