# Supplementary material for: Individual- and Group-Level Disparities Between Racial and Ethnic Groups in Lung Cancer Screening Eligibility Criteria
Source: JAMA Netw Open. 2025 Mar 27;8(3):e252172. doi: 10.1001/jamanetworkopen.2025.2172 (PMC11950895; doi:10.1001/jamanetworkopen.2025.2172)
Supplement: Supplement 1. — eMethods. eResults. eTable 1. The Percentage of Individuals Aged 50-80 Years Who Ever Smoked Who Would Be Eligible for Screening Under USPSTF-2021 Lung Cancer Screening Eligibility Guidelines, Life-Years Gained Sensitivity for Individuals Eligible Under USPSTF-2021, and the Efficiency of Screening Individuals Eligible Under USPSTF-2021, by Self-Reported Race and Ethnicity eTable 2. Mean, 25th, 50th and 75th Percentile of Life-Years Gained From Attending Screening, Estimated by the LYFS-CT Model, by Self-Reported Race And Ethnicity eTable 3. The LYFS-CT Submodels for Lung Cancer Death and Overall Mortality eTable 4. Characteristics of Hypothetical Individuals Who Are High Risk and/or High Benefit but Not Eligible for Lung Cancer Screening Under USPSTF-2021, or Low Risk and/or Low Benefit and Are Eligible for Lung Cancer Screening Under USPSTF-2021 eReferences. [file jamanetwopen-e252172-s001.pdf]

Supplementary Online Content

Young CD, Katki HA, Cheung LC, et al. Individual- and group-level disparities between racial and ethnic groups in lung cancer screening eligibility criteria. *JAMA Netw Open.* 2025;8(3):e252172. doi:10.1001/jamanetworkopen.2025.2172

eMethods.

eResults.

eTable 1. The Percentage of Individuals Aged 50-80 Years Who Ever Smoked Who Would Be Eligible for Screening Under USPSTF-2021 Lung Cancer Screening Eligibility Guidelines, Life-Years Gained Sensitivity for Individuals Eligible Under USPSTF-2021, and the Efficiency of Screening Individuals Eligible Under USPSTF-2021, by Self-Reported Race and Ethnicity

eTable 2. Mean, 25th, 50th and 75th Percentile of Life-Years Gained From Attending Screening, Estimated by the LYFS-CT Model, by Self-Reported Race And Ethnicity

eTable 3. The LYFS-CT Submodels for Lung Cancer Death and Overall Mortality

eTable 4. Characteristics of Hypothetical Individuals Who Are High Risk and/or High Benefit but Not Eligible for Lung Cancer Screening Under USPSTF-2021, or Low Risk and/or Low Benefit and Are Eligible for Lung Cancer Screening Under USPSTF-2021

eReferences.

This supplementary material has been provided by the authors to give readers additional information about their work.

© 2025 Young CD et al. *JAMA Network Open.*

eMethods.

Life-Years Gained from Screening-CT (LYFS-CT) model

LYFS-CT models the life-years gained from attending an NLST-like (3 annual low-dose CT screens) lung screening programme; for full details, see Cheung et al.[1]. This gives a measure of the *benefit* from attending screening; since risk increases with age and smoking intensity, many high-risk individuals may have limited life-expectancy and therefore only have minimal gains from attending screening due to their high risk of competing mortality.

LYFS-CT is composed of two submodels: one for individualized risk of lung-cancer death, and the other for risk of all-cause mortality. The submodel for risk of lung cancer death in the absence of CT screening is the previously validated Lung Cancer Death Risk Assessment Tool (LCDRAT) [2]. The submodel for risk of all-cause mortality was a Cox model that used the lung cancer risk factors in LCDRAT (except for family history of lung cancer), but also included 12 comorbidities highly predictive of mortality. This model was trained on NHIS data and independently validated with different NHIS data.[3] See Supplementary Table 3 for LYFS-CT model parameters.

Multiple imputation in the National Health Interview Survey

We used 5 imputations in our multiple imputation analyses, imputing data on body mass index (BMI, missing for 3.0% of individuals aged 50-80 who ever smoked), race and ethnicity (1.8% missing), the number of cigarettes smoked per day missing for 7.7% of individuals aged 50-80 who ever smoked), and the number of years since quitting smoking (missing for 0.7% of individuals aged 50-80 who formerly smoked).

For the variables BMI, race and ethnicity, and quit-years, the value to impute was randomly selected from observed values in the 2015 NHIS among individuals aged 18-85+, conditional on their age (in deciles) and gender. For the number of cigarettes smoked per day, the value to impute was selected based on additionally conditioning on smoking status (current vs former).

© 2025 Young CD et al. *JAMA Network Open.*

eResults.

High risk and high benefit individuals who are not eligible for screening under USPSTF-2021

Previous research has shown that many high risk and high benefit individuals are not eligible for lung cancer screening under USPSTF-2021[1,4-6]. In **eTable 4** we highlight some examples of hypothetical individuals who would be high risk or high benefit who are not eligible under USPSTF-2021, along with some low risk or low benefit individuals who are eligible under USPSTF-2021.

© 2025 Young CD et al. *JAMA Network Open.*

eTable 1. The Percentage of Individuals Aged 50-80 Years Who Ever Smoked Who Would Be Eligible for Screening Under USPSTF-2021 Lung Cancer Screening Eligibility Guidelines, Life-Years Gained Sensitivity for Individuals Eligible Under USPSTF-2021, and the Efficiency of Screening Individuals Eligible Under USPSTF-2021, by Self-Reported Race and Ethnicity

	African American	Asian American	Hispanic American	Non-Hispanic white
Percent eligible	26.8%	22.1%	18.9%	35.5%
LYG sensitivity	48.5%	45.2%	37.0%	64.5%
NNS/10LYG	158.7	287.9	322.2	194.8

NNS/10LYG: number needed to screen per 10 life-years gained.

© 2025 Young CD et al. *JAMA Network Open.*

eTable 2. Mean, 25th, 50th and 75th Percentile of Life-Years Gained From Attending Screening, Estimated by the LYFS-CT Model, by Self-Reported Race And Ethnicity

	Mean	25th percentile	50th percentile	75th percentile	Percentage with ≥16.2 days of life gained under LYFS-CT
African American	12.73	4.84	8.69	16.87	26.6%
Asian American	6.20	1.94	4.01	7.79	9.3%
Hispanic American	5.79	1.89	3.86	7.54	6.8%
Non-Hispanic white	10.33	3.14	6.21	13.62	19.5%

© 2025 Young CD et al. *JAMA Network Open.*

eTable 3. The LYFS-CT Submodels for Lung Cancer Death and Overall Mortality [3]

Variable	How it's coded in the model	Overall Mortality		Lung cancer Death	
		HR	95% CI	HR	95% CI
3 annual CT screens	Binary	— [a]	—	0.796 [b]	(0.69-0.92) [b]
Age	Log term	— [c]	—	431.812	(185.059,1007.578)
Sex (female)	Binary	0.708	(0.671,0.747)	0.837	(0.736,0.950)
Self-reported race/ethnicity	Categorical				
	White, non-Hispanic	1.000	Reference	1.000	Reference
	Black, non-Hispanic	1.294	(1.195,1.402)	1.482	(1.176,1.869)
	Hispanic	0.812	(0.734,0.899)	0.687	(0.397,1.190)
	Asian or Other	0.619	(0.493,0.778)	0.657	(0.447,0.964)
Education [d]	Trend	0.930	(0.915,0.944)	0.908	(0.873,0.944)
Calendar year assessed	Linear	0.977	(0.969,0.985)	—	—
BMI≤18.5	Binary	—	—	1.428	(0.899,2.268)
BMI	Log term	—	—	0.447	(0.296,0.675)
	Categorical			—	—
	20<-25	1.000	Reference		
	≤18.5	1.879	(1.631,2.166)		
	18.5<-20	1.370	(1.219,1.539)		
	25<-30	0.793	(0.747,0.842)		
	30<-35	0.815	(0.755,0.880)		
	>35	0.980	(0.894,1.074)		
Pack years	Categorical	—	—		
	0-<30			1.000	Reference
	30-<40			1.743	(1.354,2.244)
	40-<50			2.112	(1.679,2.657)
	50+			2.446	(1.861,3.214)
	Sqrt term	1.081	(1.053,1.110)		

© 2025 Young CD et al. *JAMA Network Open.*

Quit years	Log term [e]	0.839	(0.818,0.860)	0.686	(0.640,0.735)
Years smoked	Log term	—	—	1.395	(1.099,1.771)
>1 pack/day	Binary	—	—	1.273	(1.054,1.539)
Cigs/day	Log term [e]	0.966	(0.893,1.045)	—	—
Lung cancer family history [f]	Trend [g]	—	—	1.525	(1.300,1.789)
Use special equipment [h]	Binary	1.748	(1.624,1.881)	—	—
Liver condition, past 1y	Binary	1.650	(1.416,1.923)	—	—
Emphysema	Binary	1.615	(1.491,1.749)	1.741	(1.450,2.090)
Diabetes	Binary	1.518	(1.422,1.620)	—	—
Weak/failing kidneys, past 1y	Binary	1.475	(1.288,1.689)	—	—
Prior cancer	Binary	1.322	(1.244,1.405)	—	—
Prior stroke	Binary	1.319	(1.212,1.436)	—	—
Prior heart attack	Binary	1.292	(1.187,1.412)	—	—
Coronary heart disease	Binary	1.182	(1.080,1.294)	—	—
Heart disease	Binary	1.143	(1.073,1.218)	—	—
Chronic bronchitis, past 1y	Binary	1.130	(1.037,1.232)	—	—
Hypertension	Binary	1.118	(1.066,1.173)	—	—
Angina pectoris	Binary	0.912	(0.829,1.004)	—	—

Abbreviations: BMI= Body mass index; CI= Confidence interval; 1y = 1 year; CT= Computed tomography; HR = Hazard Ratio; LCDRAT= Lung cancer Death Risk Assessment Tool.

[a] "—" indicates that the specified risk factor/parameterization was not included in the model.
[b] Represents the rate-ratio observed in the NLST over 5 years of follow-up.
[c] Age is used as the time scale for the overall mortality model.
[d] <12 grade=1, high-school graduate=2, post high-school but no college=3, some College=4, Bachelor's degree=5, graduate school=6.
[e] Natural logarithm of 1+quit-years
[f] FDR = First-degree relatives (siblings, parents, children) with history of lung cancer.
[g] Definition: No FDRs with lung cancer = 0, 1 FDR with lung cancer = 1, Two or more FDRs with lung cancer = 2.
[h] Have health problems that require use of special equipment, such as a cane, a wheelchair, a special bed, or a special telephone.

© 2025 Young CD et al. *JAMA Network Open.*

eTable 4. Characteristics of Hypothetical Individuals Who Are High Risk and/or High Benefit but Not Eligible for Lung Cancer Screening Under USPSTF-2021, or Low Risk and/or Low Benefit and Are Eligible for Lung Cancer Screening Under USPSTF-2021

	A	B	C	D	E	F
Age	73	62	79	50	51	55
Sex	Male	Male	Female	Male	Male	Female
Self-reported race and ethnicity	Non-Hispanic white	African American	African American	African American	Asian American	Non-Hispanic white
Education	≤12th grade	High school graduate	≤12th grade	High school graduate	Bachelors degree	High school graduate
BMI	25.1	24.7	23.3	35.2	20.8	21.8
Pack-years	58.5	11.75	15.25	30	21.75	28
Quit-years	20	0	0	5	0	12
Smoke-years	39	47	61	30	29	28
Cigarettes per day	30	5	5	20	15	20
Family history of lung cancer	Yes	Yes	No	No	No	No
Additional conditions:	Hypertension	Hypertension	Hypertension, coronary heart disease,	Hypertension, stroke, uses special equipment	None	None
USPSTF-2021 eligibility	No	No	No	Yes	Yes	Yes
Lung cancer death risk*	4.1%	2.4%	5.0%	0.2%	0.2%	0.2%
Benefit from screening**	45 days	31 days	54 days	4 days	4 days	4 days

© 2025 Young CD et al. *JAMA Network Open.*

eReferences.

1. Cheung LC, Berg CD, Castle PE, Katki HA, Chaturvedi AK. Life-Gained–Based Versus Risk-Based Selection of Smokers for Lung Cancer Screening. *Ann Intern Med*. 2019;Nov 5;171(9):623-632.2019
2. Katki HA, Kovalchik SA, Berg CD, Cheung LC, Chaturvedi AK. Development and Validation of Risk Models to Select Ever-Smokers for CT Lung Cancer Screening. *JAMA*. Jun 07 2016;315(21):2300-11. doi:10.1001/jama.2016.6255
3. Cheung LC, Berg CD, Castle PE, Katki HA, Chaturvedi AK. Life-Gained-Based Versus Risk-Based Selection of Smokers for Lung Cancer Screening. *Annals of internal medicine*. Nov 5 2019;171(9):623-632. doi:10.7326/M19-1263
4. Zhang EY, Cheung LC, Katki HA, Graubard BI, Jemal A, Chaturvedi AK, Landy R. The risk and benefit profiles of US-eligible lung cancer screening attendees vs nonattendees. *JNCI: Journal of the National Cancer Institute*. 2024;116(12):1896-1903.
5. Choi E, Ding VY, Luo SJ, et al. Risk model–based lung cancer screening and racial and ethnic disparities in the US. *JAMA oncology*. 2023;9(12):1640-1648.
6. Aredo JV, Choi E, Ding VY, et al. Racial and ethnic disparities in lung cancer screening by the 2021 USPSTF guidelines versus risk-based criteria: the multiethnic cohort study. *JNCI Cancer Spectrum*. 2022;6(3):pkac033.

© 2025 Young CD et al. *JAMA Network Open.*
