# Supplementary material for: Individual- and Group-Level Disparities Between Racial and Ethnic Groups in Lung Cancer Screening Eligibility Criteria
Source: JAMA Netw Open. 2025 Mar 27;8(3):e252172. doi: 10.1001/jamanetworkopen.2025.2172 (PMC11950895; doi:10.1001/jamanetworkopen.2025.2172)
Supplement: Supplement 2. — Data Sharing Statement [file jamanetwopen-e252172-s002.pdf]

Data Sharing Statement

Young. Individual- and Group-Level Disparities Between Racial and Ethnic Groups in Lung Cancer Screening Eligibility Criteria. *JAMA Netw Open*. Published March 27, 2025. doi:10.1001/jamanetworkopen.2025.2172

Data
Data available: Yes
Data types: Deidentified participant data, Data dictionary
How to access data: Data publicly available at https://www.cdc.gov/nchs/nhis/nhis_2015_data_release.htm.
When available: beginning date: 08-18-2024

Supporting Documents
Document types: None

Additional Information
Who can access the data: Data available online without limitations.
Types of analyses: Any purpose.
Mechanisms of data availability: Data available online without limitations.
